# Missed opportunities in early psychosis care: retrospective chart review of cardiovascular disease monitoring, disengagement and weight changes in a Ghanaian psychiatric hospital

**DOI:** 10.1192/bji.2025.7

**Published:** 2025-08

**Authors:** Joel Agorinya, Emelda Edem Adzo Asem-Ahiablee, Emefa Adzo Dzordzorme, Jude Nazir Ayambila, Elsie Agyeman Amaning, Chukwuebuka Ohakpougwu, Abdulai Rafiq Arhin, Kwadwo Marfo Obeng, Belinda Lennox

**Affiliations:** 1 Accra Psychiatric Hospital, Accra, Ghana; 2 Department of Psychiatry, University of Oxford, Oxford, UK

**Keywords:** Psychosis, antipsychotics, disengagement, cardiovascular risk, weight gain

## Abstract

**Background:**

Patients with psychosis face an elevated risk of cardiovascular mortality and are more likely to disengage from care. While antipsychotics are essential for treatment, they further increase this risk. Despite this, Ghana lacks a national policy for monitoring cardiovascular risk factors in individuals on antipsychotics.

**Aims:**

To evaluate disengagement in care and weight changes among newly diagnosed psychotic patients at Accra Psychiatric Hospital, and to inform clinical practice.

**Method:**

A retrospective review of medical records was conducted for patients newly diagnosed with non-affective psychotic disorders between June 2022 and May 2023. Patients were reviewed for 6 months, with assessments at baseline, 3 months and 6 months. Outcomes included antipsychotic prescription patterns, dropout rates, cardiovascular disease monitoring and weight changes. Descriptive statistics, multinomial logistic regression and linear mixed-effects models were used for analysis.

**Results:**

The number of patients disengaged from care within the first month was 53.1%, and within 6 months 75.5%; 62.8% received olanzapine at baseline. Weight gain was exponential, with 40% experiencing clinically significant weight gain at 3 months, increasing to 58% at 6 months. Less than 50% of patients had their blood sugar and lipid profiles checked before starting antipsychotics. Higher baseline weight was associated with increased weight over time (*β* = 0.96, *t* = 80, *P* < 0.001, 95% CI 0.93, 0.98).

**Conclusions:**

High disengagement rates, low cardiovascular disease monitoring and exponential weight gain were observed. Targeted interventions, robust monitoring protocols and further research are needed to improve patient outcomes.

An estimated 4% of the world’s population experiences psychosis^1^ – mental health symptoms characterised by loss of contact with reality that may manifest as delusions, hallucinations or disorganised thoughts or behaviours.^2^ Psychotic disorders pose significant challenges to individuals, their families and society because they are associated with productivity losses, morbidity and mortality, including suicidality.^3,4^ Effective treatment of psychosis necessitates engagement with the formal healthcare system for pharmacological and psychosocial interventions. However, patients experiencing psychosis are often deterred from seeking care due to stigma, religious/cultural beliefs or financial constraints.^5^ In Ghana, psychotic disorders are the mental disorders most diagnosed, probably due to the disruptive nature of severe psychotic symptoms, and account for most psychiatric in-patients.^6^ These patients probably represent a small proportion of cases with the most severe symptoms, because many individuals experiencing psychosis in Ghana do not engage with the formal health system.^7^ This situation is exacerbated by inadequate mental healthcare investment, a shortage of skilled professionals and the influence of religious and cultural beliefs that contribute to delays in seeking care.^7^

## Disengagement from care

The systemic issues not only hinder initial access to mental health services but also contribute to ongoing patient disengagement. Even after accessing mental health services, patients often disengage due to cost, lack of support, poor insight or stigma.^8^ Disengagement from care worsens symptoms and disrupts the lives of patients and their families. Engaging patients in the early stages of psychosis is critical to minimising its impact and improving outcomes.^9^

## Impact of antipsychotics on cardiovascular outcomes

Individuals with psychosis have a reduced life expectancy of 10–15 years,^10^ with cardiovascular disease being a major contributor to this excess mortality.^11^ Metabolic syndrome, which is exacerbated by clinically significant weight gain (CSWG) (≥7% of baseline weight),^12,13^ increases the risk of cardiovascular disease and mortality.^14^ Unfortunately, antipsychotics, although necessary for treating psychosis, contribute to weight gain and metabolic issues through multiple mechanisms.^15^ This risk is highest in the early stages of treatment.^16^ Recent systematic reviews and meta-analyses have shown that most antipsychotics cause weight gain;^17,18^ the difference, however, lies in the magnitude of the weight gain and the proportion of patients who experience this outcome. This suggests that there are really no ‘safe antipsychotics’ in regard to weight gain.

Special monitoring of cardiovascular disease risk factors is therefore vital for extending the lifespan of patients with psychosis. Guidelines from both Europe and the USA recommend frequent monitoring of fasting blood sugar and lipid profiles, especially during the initial months of antipsychotic treatment.^19^ Given that the treatment options available may not significantly reverse antipsychotic-induced weight gain and metabolic abnormalities,^20^ prevention through lifestyle modifications and early detection via regular testing are crucial. There are no equivalent protocols for cardiometabolic monitoring for people with psychosis in Ghana.

## Current situation in Ghana and Sub-Sahara Africa

Accra Psychiatric Hospital is the oldest psychiatric hospital in Ghana and one of the oldest in West Africa, serving as the primary training centre for mental healthcare in that country. It is the main centre for training of psychiatrists under both the Ghana and West Africa Colleges of physicians in Ghana. In addition, many medical schools and allied health programmes in Ghana receive mental health training from the Accra Psychiatric Hospital. The hospital has a capacity of 300 beds, with acute and chronic wards and a residential drug rehabilitation centre. It also has a medical laboratory, clinical psychology, occupational therapy, social welfare and community psychiatry units. It attends to over 600 new cases of non-affective psychosis annually.

One study in Nigeria found that the mean body mass index (BMI, kg/m^2^) of patients prescribed second-generation antipsychotics increased from 23.7 at baseline to 31 after 3 months.^21^ Luckhoff et al^22^ also found that BMI change scores inversely correlated with Positive and Negative Syndrome Scale scores in a South African population. However, these studies did not estimate the proportion of patients who became overweight or obese over time. There is a gap in the knowledge regarding the effects of antipsychotics on the weight and BMI of patients taking antipsychotics in Ghana. Additionally, there is little information regarding the characteristics of psychotic patients in Ghana that predispose them to antipsychotic-induced weight gain. The absence of both guidelines on the prescription of antipsychotics and monitoring for cardiovascular disease lead to delay in detecting patients that require treatment.

## Current study

We aimed to address this gap in knowledge at Accra Psychiatric Hospital, by reviewing patients’ attendance patterns and cardiovascular risk (trends in weight and BMI) among patients newly diagnosed with psychosis for the first 6 months following initial engagement in care. We also examined some correlates of cardiovascular disease risk factors. Overall, we aim to evaluate disengagement in care and cardiovascular disease risk outcomes among newly diagnosed psychotic patients at Accra Psychiatric Hospital.

## Method

### Sample and setting

This is a retrospective cohort study conducted by reviewing the anonymised electronic medical records of patients at Accra Psychiatric Hospital in Ghana. The inclusion criteria were all patients newly diagnosed with non-affective psychotic disorder according to the ICD-10^23^ criteria (F20–29) during the study period (1 June 2022 to 31 May 2023) and who had received antipsychotic medication. We included both voluntary and involuntary patients. Patients under the age of 18 years were excluded in the analysis of weight and BMI changes but were included in the analysis of disengagement and medication prescription patterns at first visit. All patients were followed for a maximum of 6 months, with assessments at baseline, 3 months and 6 months. The 6-month cut-off point was used because, beyond that time point, the disengagement rate was close to 100%. Ethical approval was obtained from Accra Psychiatric Hospital’s Research and Ethics Committee (no. APH-ERC 00012/23). Six research assistants performed data abstraction after receiving training on the protocol and data abstraction tool. The principal investigator audited the work to ensure compliance with the protocol.

### Outcome measures

Outcome variables collected included medication choice, dropout status in the first 6 months, evidence of cardiovascular disease monitoring and weight changes. We also collected demographic information (age, sex, occupation) and anthropometric data (weight [kg] and BMI/ [weight/height]),^2^ which were used as covariates in our analysis. The data were clustered at baseline, 3 ± 1 months and 6 ± 1 months. Where there were multiple visits, the last before the particular time point was used. All weights were recorded using the same weighing scale at the hospital outpatient department.

### Statistical analysis

Statistical analysis was performed using R version 4. Descriptive statistics, including frequencies (percentages) and means (±s.d.) were used to analyse categorical and continuous baseline data, respectively. The prescription pattern and factors influencing the choice of medication were also analysed, using multinomial logistic regression. We further analysed the pattern and risk factors for weight change using a linear mixed-effects model. The fixed effects in this model were patients’ age, baseline weight, sex, time and medication. In comparing weight change based on medication choice, olanzapine was set as the reference medication because it was the medication prescribed most often. Medications with frequency below ten (chlorpromazine, clozapine, paliperidone and quetiapine) were excluded from the analysis. Results from the models include 95% CIs to provide estimates of precision. Missing data were omitted during the analysis.

## Results

### Baseline characteristics and prescribed medication

Table 1 shows the mean age, weight, BMI and prescribed antipsychotic of patients at their first visit to the study site.

**Table 1:** Baseline characteristics and prescribed medication

	Male	Female
*N* (%)	278 (50.1)	277 (49.9)
Mean age, years (± s.d.)	33.6 (12.5)	40.4 (14.3)
Mean baseline weight, kg (± s.d.)	63.7 (14.8)	60.2 (16.4)
Mean baseline BMI, kg/m2 (± s.d.)	21.3 (4.53)	23.3 (6.05)
Baseline BMI category, %		
Underweight	28.3	19.6
Normal weight	53.4	45.7
Overweight/obese	18.3	34.7
Antipsychotic, %		
Aripiprazole	5.8	2.0
Depot	7.3	4.5
Haloperidol	3.1	1.0
Olanzapine	64.9	60.8
Risperidone	18.8	31.7

### Hospital attendance

**Fig. 1:**
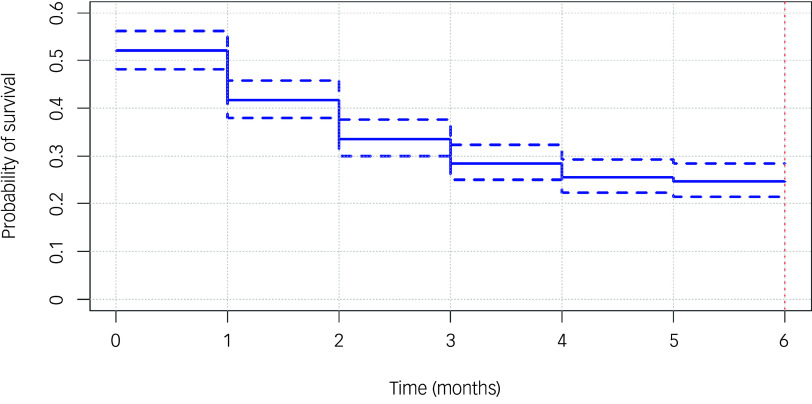
Probability of disengagement within the first 6 months. Survival curve showing the probability of patients dropping out at various time points (months) within the first 6 months.

The majority of patients (75.5%) stopped attending reviews within the first 6 months. More than half (53.1%) of patients did not engage with the service for up to 1 month; they either never attended again after their first visit or were seen within 1 month of their first visit and then became lost to follow-up. Most patients (39%) did not return for review following their first visit, while only 24% and 14% attended a total of one and two additional reviews, respectively, after the first visit. Figure 1 shows the probability of disengagement at various time points within the first 6 months of care.

### Cardiometabolic monitoring

From our analysis, olanzapine was the antipsychotic most prescribed (62.8%) and aripiprazole the least (3.6%). Despite the high rate of prescribing olanzapine, less than half of the patients at their first visit had their blood sugar (43%) or lipid profile (26%) requested/recorded before initiating antipsychotic treatment. This pattern did not improve over time, with under 5% having had their blood sugar or lipid profile tested at each subsequent visit. Furthermore, 13% of patients did not have records of their weight at first visit. A multinomial logistic regression using patients’ age, sex and baseline weight as predictors indicated that patients with higher baseline weight were more likely to receive aripiprazole (odds ratio 1.07, 95% CI 1.04, 1.10, *P* < 0.001), haloperidol (odds ratio 1.05, 95% CI 1.01, 1.09, *P* = 0.03) or risperidone (odds ratio 1.04, 95% CI 0.07, 1.05, *P* < 0.001) in comparison with olanzapine.

### Weight change over time

At 3 months, 40% of patients experienced clinically significant weight gain (≥7% of baseline weight), and this increased to 58% at 6 months.

**Fig. 2:**
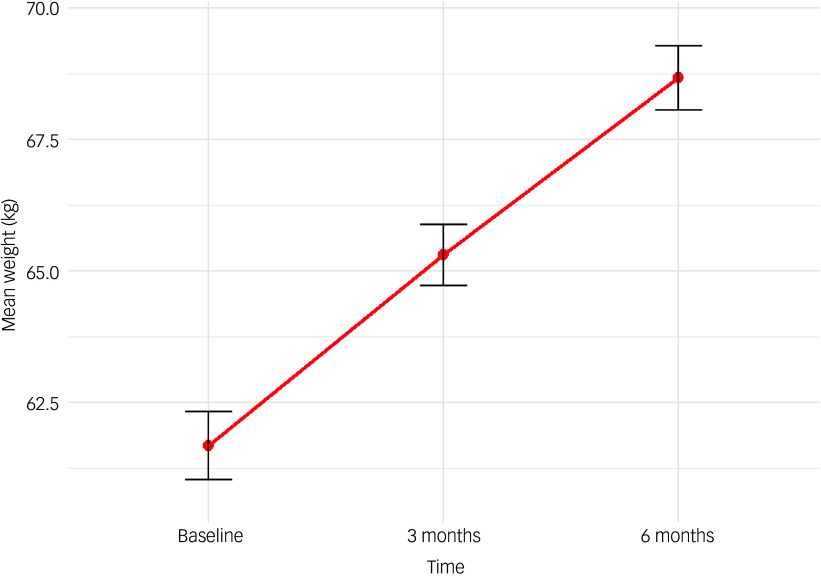
Weight change in the first 6 months. Line chart showing the mean weight trajectory of patients in the first 6 months of starting antipsychotics.

We fitted a linear mixed-effects model to examine the effects of selected covariates on weight change, incorporating age, sex, baseline weight, medication type and time, with a random effect for individual subjects. The results (Table 2) indicated that higher baseline weight was associated with significantly greater weight over time (*β* = 0.96, *P* < 0.001). Additionally, weight increased significantly over the 6-month follow-up period, with notable weight gains of 8.6% above baseline weight at 3 months (*β* = 3.33, *P* < 0.001) and increasing to 12.2% at 6 months (*β* = 6.16, *P* < 0.001), as illustrated in Fig. 2. None of the medication types, when compared with olanzapine, showed statistically significant different effects on weight change. Additionally, sex was not a significant predictor of weight change (*β* = 0.39, *P* = 0.264).

**Table 2:** Effects of various covariates on weight change

Parameter	Coefficient	s.e.	*t*-value	*P*-value	95% CI
Intercept	61.61	0.30	205.03	<0.001***	[61.02, 62.20]
Age	−0.02	0.01	−1.61	0.108	[−0.04, 0]
Baseline weight	0.96	0.01	80.06	<0.001***	[0.93, 0.98]
Sex	0.39	0.35	1.12	0.264	[−0.29, 1.07]
Aripiprazole	−0.95	0.81	−1.16	0.244	[−2.54, 0.65]
Depot	0.86	0.75	1.14	0.254	[–0.62, 2.33]
Haloperidol	−1.61	1.30	1.24	0.216	[−4.17, 0.94]
Risperidone	−0.43	0.40	−1.07	0.284	[−1.22, 0.36]
3 months	3.33	0.34	10.07	<0.001***	[2.76, 4.10]
6 months	6.18	0.51	12.16	<0.001***	[5.19, 7.18]

*** *P* < 0.01.

### Change in BMI

Patients’ BMI was dichotomised as either not overweight (BMI <25) or overweight (BMI ≥25) and was followed over time. BMI values generally increased over time in a way similar to weight. At baseline, about one quarter of patients (25.9%) had BMI ≥25 and, at 6 months, half of those with records available (52%) were overweight.

## Discussion

This study used the medical records of patients at Accra Psychiatric Hospital in Ghana to analyse the pattern of attendance, antipsychotic prescription practices and weight and BMI changes over the first 6 months of treatment. Our results demonstrate a significant service disengagement rate, poor cardiometabolic risk monitoring and significant changes in weight and BMI over the first 6 months.

### Baseline characteristics

The average age of our study population (37.4 years) is generally higher than findings from other countries in Africa and the UK.^24,25^ This higher age of presentation probably reflects the prolonged duration of untreated psychosis in low- and middle-income countries, and the multiple pathways to care in Ghana.^26^ Additionally, because Accra Psychiatric Hospital is a referral centre, there may be delays between the onset of psychosis and hospital presentation.

The mean BMI of 22.2 kg/m2, with 25.9% of patients being either overweight or obese, is comparable to the general Ghanaian population mean BMI of 24.7 kg/m2.^27^

### Disengagement from service

Psychotic disorders generally follow a chronic course, usually requiring prolonged treatment. It is recommended that a first-episode psychosis be treated until symptoms have been in remission for a minimum of 1 year, to prevent exacerbations.^28^ Our finding that over half (53.1%) of patients with non-affective psychosis disengaged within 1 month, and 75.5% by 6 months, is alarming. This rate is significantly higher compared with other regions,^29^ highlighting a particular issue in Ghana.

Several factors may have contributed to this high disengagement rate. Patients may transfer to community mental health services due to proximity, although this is unlikely to account for most cases because standard practice requires that patients be clinically stable before transfer to community care. Another factor is resorting to religious and faith healers, especially prayer camps, which is common and widely practised in Ghana.^30^ During the period under this review, patients and their caregivers had to bear the cost of care for mental health services, and this is another possible explanation for the high rates of disengagement. Additionally, some patients may have achieved full recovery and returned to baseline functioning, reducing their perceived need to continue to engage with mental health services, with some evidence suggesting that patients with early psychosis show good treatment response.^31^ Furthermore, our review included all patients diagnosed with a non-affective disorder, and this includes patients with acute and transient psychotic disorders. These disorders are known to resolve quickly, usually within 1 month,^2^ and these patients are unlikely to feel the need to return for review, especially considering the stigma associated with a psychiatric diagnosis. It is now a priority to understand the reasons for patient disengagement, to help to develop strategies aimed at improving retention and treatment outcomes.

### Cardiometabolic considerations

Despite growing evidence concerning the metabolic consequences of olanzapine,^32^ 63% of patients in our review received this medicatiion during their first visit. Clinicians probably prefer olanzapine due to its perceived superior efficacy in resolving psychotic symptoms,^33^ prioritising immediate clinical response over the risk of long-term metabolic dysregulation. Other countries recommend that olanzapine not be used as a first-line treatment for psychosis because of its metabolic profile.^34^ The low levels of use of long-acting injectable (LAI) antipsychotics (depot) in our study population could represent a cost issue. While second-generation antipsychotics are the preferred first-line treatment, LAIs are expensive and many patients cannot afford them. Clinicians might opt against first-generation LAIs due to their extrapyramidal side-effects.

Proactive prevention of cardiovascular-related mortality requires regular monitoring of cardiometabolic risk factors. The Royal College of Psychiatrists recommends cardiometabolic tests to monitor physical health in the early stages of antipsychotic treatment, including weight, BMI, blood pressure, plasma glucose and lipid profiles.^35^ Our study’s finding of low levels of cardiometabolic monitoring, even among those receiving olanzapine, means that these standards are not being met, exposing patients to risk. Under half of patients had their blood sugar levels recorded before initiating antipsychotics, and this proportion did not improve significantly over time. Similarly, lipid profile monitoring was insufficient. The absence of established local protocols for cardiometabolic monitoring probably contributes to this issue, as well as the cost of laboratory investigations, which are borne by the patient or their caregiver. However, this cannot explain the failure to record patients’ weight during initial consultation.

### Weight and BMI changes over time

Patients demonstrated progressive weight and BMI increases over the course of this review, consistent with findings from other studies in patients with first-episode psychosis.^36^ People with higher baseline weight continued to record further increases.

Our analysis revealed that weight change associated with the other medications was not different from that of olanzapine. This finding of insignificant difference in the contribution of medication type to weight changes conflicts with the existing literature, which typically shows olanzapine to be associated with the greatest weight gain.^36,37^ The lack of randomisation in our study may have introduced selection bias based on clinician preferences, influencing the observed effects on weight changes. Olanzapine was preferentially prescribed for patients with lower baseline weight and, because lower baseline weight is associated with less weight gain in our population, people on other medications are likely to have differentially gained more weight, potentially masking the effects of olanzapine on weight. This would be plausible given that almost all antipsychotics are known to cause weight gain.^17,18,38^ However, this could also be a type II error, with a lack of power to detect differences between medications. Our finding that 40% of patients experienced CSWG at 3 months and 58% at 6 months is higher than those of a recent meta-analysis on the association between antipsychotics and CSWG.^17^ At baseline, one in four patients was either overweight or obese and, by 6 months, this proportion had increased to one in two. Research has pointed to racial differences in the experience of antipsychotic-induced weight gain with people of black ancestry recording higher weight gains than their caucasian counterparts.^39^ Genetic variability in the expression of antipsychotic target genes and antipsychotic metabolism has been proposed as a reason for these observed racial disparities,^40,41^ and this could be the reason why our study population, which is entirely African, recorded higher rates of weight increase. This calls for early, proactive efforts when initiating antipsychotics to prevent weight gain, identify those gaining weight and initiate measures to mitigate the impact on their physical health. Because there are no established guidelines for dealing with antipsychotic-induced weight gain, implementation of mitigation measures is at the discretion of the clinician. Having guidelines allows the hospital to plan and make resources for lifestyle or pharmacological interventions available for all patients.

### Future research directions

This study highlights significant patient disengagement and lapses in both cardiometabolic risk monitoring and weight management in patients with psychosis at Accra Psychiatric Hospital. Even though feedback has been provided to the hospital, addressing these challenges requires robust monitoring protocols to ensure that patients’ cardiovascular risks are routinely evaluated, especially in the first few months of initiating antipsychotic treatment. Evidence-based interventions should be recommended to mitigate the cardiovascular risks associated with exposure to antipsychotics, and these should be timely, adopting both preventive and therapeutic approaches. There is also a need for ongoing research to understand the factors contributing to the high disengagement rate, and to develop interventions to address this problem. While these findings are restricted to the study site, the contributory factors are common to most low- and middle-income countries and these findings are likely to be replicated in similar settings.

### Study limitations

This study utilised real-world clinical data, with significant missing data, which is a limitation to its interpretation. Additionally, the absence of randomisation potentially introduces bias in the allocation of medications, thus affecting the interpretation of the relationship between antipsychotic type and weight/BMI changes. Furthermore, there are probably unmeasured confounding variables such as genetic, lifestyle and dietary factors that could potentially account for a significant proportion of the variation noted in weight and BMI. Finally, because our study was a retrospective review, we were unable to obtain comprehensive follow-up data to ascertain the cause of some findings, such as the high disengagement rate.

## Data Availability

The data that support the findings of this study are available from the corresponding author, J.A., upon reasonable request.
